# An intra-population analysis of the indris’ song dissimilarity in the light of genetic distance

**DOI:** 10.1038/s41598-017-10656-9

**Published:** 2017-08-31

**Authors:** Valeria Torti, Giovanna Bonadonna, Chiara De Gregorio, Daria Valente, Rose Marie Randrianarison, Olivier Friard, Luca Pozzi, Marco Gamba, Cristina Giacoma

**Affiliations:** 1 Department of Life Sciences and Systems Biology (DBIOS), University of Torino, Torino, Italy; 2 Département de Paléontologie et d’Anthropologie Biologique, Faculté des Sciences, Université d’Antananarivo, Antananarivo, Madagascar; 3 Groupe d’étude et de recherche sur les primates de Madagascar (GERP), Antananarivo, Madagascar; 4 Department of Anthropology, The University of Texas at San Antonio, One UTSA Circle, San Antonio, TX 78249 United States of America

## Abstract

The increasing interest in the evolution of human language has led several fields of research to focus on primate vocal communication. The ‘singing primates’, which produce elaborated and complex sequences of vocalizations, are of particular interest for this topic. Indris (*Indri indri*) are the only singing lemurs and emit songs whose most distinctive portions are “descending phrases” consisting of 2-5 units. We examined how the structure of the indris’ phrases varied with genetic relatedness among individuals. We tested whether the acoustic structure could provide conspecifics with information about individual identity and group membership. When analyzing phrase dissimilarity and genetic distance of both sexes, we found significant results for males but not for females. We found that similarity of male song-phrases correlates with kin in both time and frequency parameters, while, for females, this information is encoded only in the frequency of a single type. Song phrases have consistent individual-specific features, but we did not find any potential for advertising group membership. We emphasize the fact that genetic and social factors may play a role in the acoustic plasticity of female indris. Altogether, these findings open a new perspective for future research on the possibility of vocal production learning in these primates.

## Introduction

Vocal signals often play a critical role in animal communication^1^. While many species make a conspicuous use of vocalizations, a limited number of taxa communicate using a sequence of vocal emissions, usually termed songs^2^. A song is a combination of different components that can be described hierarchically. Individual sounds are referred to as ‘units’, ‘elements’ or ‘notes’. One or more units that occur together can be called song ‘syllables’, and a sequence of one or more syllables is described as either a ‘phrase’ or ‘motif’. Since Darwin, scholars have been interested in understanding whether the complexity of singing is genetically determined, impacted by social experience, or the result of a learning process^3, 4^. The processes leading to song diversity have been widely investigated in birds. Endogenous and exogenous factors modulate the interplay between genetic characteristics, social experience, and learning^5^. For instance, early findings on song development in the zebra finches (*Taeniogypta guttata*) suggested that, while male birds develop their song during a sensitive period for vocal production learning^6^, song culture appears as a multi-generational phenotype, partially encoded in the genes of an isolated founding population. In this species, juvenile birds, that imitate isolated tutors, changed particular characteristics of the songs. These alterations can be accumulated over learning generations until a new natural song emerges^7^.

Although birds are the only animals in which vocal production learning has been rigorously associated with the modification of the cerebral connections, previous research showed that learning in vocal communication is not unique to them. Some terrestrial and marine mammals may possess the ability to learn the production of particular emissions^8^, and individuals of other species can learn the context in which to produce a particular call or how to modify their response to others’ vocalizations^9^. Communication using songs is widespread in different groups of birds and marine mammals, but is rare in primates^10^. A key question is to what extent primate vocalizations can be shaped by vocal production learning processes (hereafter, vocal learning) and social factors, and whether they may possess information useful for kin recognition. The current evidence is contradictory, with data suggesting both vocal learning and genetic relatedness as forces in shaping primate vocal signals. For instance, Marshall and colleagues^11^ suggested that genetic relatedness had a limited effect on the acoustic similarity in chimpanzee’s vocalizations (*Pan troglodytes*), since unrelated males showed a similar acoustic structure in their pant hoots. Further studies on chimpanzees consolidated the idea that vocal learning and convergence may play a role in the acoustic structure of food grunts shared within two groups^12^. The influence of social factors in shaping vocal signals has also been suggested for monkeys. Lemasson and colleagues found that social bonding better explained the acoustic similarity in the vocalizations of Campbell’s monkeys (*Cercopithecus campbelli*) when compared to genetic relatedness^13^. Although evidence seems to suggest that primate calls are not completely genetically determined, the question whether information about genetic relatedness, crucial for kin recognition, is retained in vocalization is still open. A study of mandrill’s vocalizations (*Mandrillus sphinx*)^14^ showed that the acoustic structure of contact calls was more similar between relatives than among unrelated individuals, suggesting that mandrill acoustic signals contain kin-specific information.

Because vocal learning has been typically associated with singing, studying the effects of genetics and social factors on the vocal output of primates that communicate using songs (i.e., tarsiers, gibbons, indris, titi monkeys^15^) is of great interest. Unfortunately, limited knowledge about the ontogenesis of primate singing behavior prevents a proper comparison with studies on birdsongs. However, previous research on gibbons reported that juveniles and young females produce immature vocalizations (*Hylobates lar* and *H. agilis* ^16^) and that the structure of the songs may reach a mature form at about six years old (*Nomascus gabriellae* ^17^). Moreover, the co-singing of mothers and daughters in *Hylobates agilis* has been interpreted as a possible form of tutoring to switch from an immature to a mature female great call^18^. On the other hand, concordance between song and genetic diversity across the crested gibbons^19^ suggests that genes may play a major role in shaping song structure. Additionally, both hybrid males and females showed intermediate song structure compared to the songs of the parental species (*Hylobates lar* x *H. muelleri* ^20^; *Hylobates lar* x *H. pileatus* ^21^). Interestingly, female songs tended to diverge more from their parents’ songs, while males’ appeared to resemble those of their father.

To further explore the processes shaping primate songs, we investigated the relationship between genetic distance and acoustic similarity in the indris. We also aimed to understand whether the acoustic structure of phrases differed when analyzed within closely (e.g.; father-offspring, mother-offspring) and distantly related indris in the population of Maromizaha.

The indri (*Indri indri* ^22^) is the only lemur that communicates through songs. The indris’ songs are long sequences of vocal units that are organized in phrases^23, 24^. They have the form of a chorus in which all the adults and the subadults of a group utter their contribution in a precise and coordinated manner^25^. Songs have various functions depending on the context in which are emitted and they are used for both inter and intra-group communication^26, 27^. Furthermore, songs are likely to provide information about the group composition and mediate the formation of new groups^25, 28, 29^. Because this species lives in familiar groups (Bonadonna, *unpublished data*) and the song has a rich repertoire of units^25, 30, 31^, the indris can be an excellent model to investigate the relationship between genetic relatedness and song similarity. Recent studies by Gamba and colleagues^25^ showed that the acoustic structure of phrases did not significantly change between age classes, suggesting that a limited variation may occur during ontogeny.

The inheritance of song characteristics has been inferred from the studies of hybrid gibbons. Geissmann^21^ studied the song of a male and a female hybrid (*Hylobates pileatus* x *H. lar*) finding that their songs differed markedly from the song characteristics of the parent species. However, studies on ground squirrels (*Spermophilus suslicus*) found a weak correlation between acoustic similarity and kinship, showing that other factors, such as the need for an individually distinctive acoustic structure, may play a critical role in vocal communication^32–34^.

In this study, we hypothesize that, if genetics strongly determines song characteristics, vocal learning may not play an important role in shaping the indris’ songs. We predicted that if song traits are mainly inherited, a high genetic distance will correspond to a reduced song similarity and that this reduction would be consistent within and between sexes. But it is also possible that emitters possess the potential for modifying their utterances and use songs to advertise their individuality and their belonging to a group^35–37^. In this second scenario, measures of genetic relatedness are not associated with song traits and we predicted that genetic distance and song similarity should not covary, but individuality and group membership would instead explain most of the acoustic variation.

## Results

### Extraction of the principal components

Four principal components accounted for 83.2% of the total variance of the temporal variables of the descending phrases of two units (DP2, see Supplementary Table S1), and five components accounted for 92.5% of the variance of frequency parameters (see Supplementary Table S2). We found six components for the temporal variables of the descending phrases consisting of three elements (DP3s) explaining 83% of the observed variance. We then found six components for the frequency variables of DP3s explaining 89.5% of the observed variance (see Supplementary Tables S3 and S4). The acoustic parameters showing the highest loadings on the principal components PC1 of each PCA were the first inter-onset interval (IOI1) and the total duration of unit 1 (Dur_unit1) for the temporal parameters of DP2. For frequency parameters of DP2, they were the average fundamental frequency of unit 2 (f0mean_unit2) and unit 1 (f0mean_unit1). For the temporal parameters of DP3, they were the second inter-onset interval (IOI2) and the duration of the second interval (Dur_int2). For the frequency parameters of DP3, they were the average fundamental frequency of unit 2 (f0mean_unit2), the frequency at the upper limit of the second quartiles of energy (Q50_unit2). A complete list of PCs and the loadings of the acoustic parameters are listed in the Supplementary Information (see Supplementary Tables S1–S4).

### Acoustic similarity and genetic relatedness

**Figure 1:**
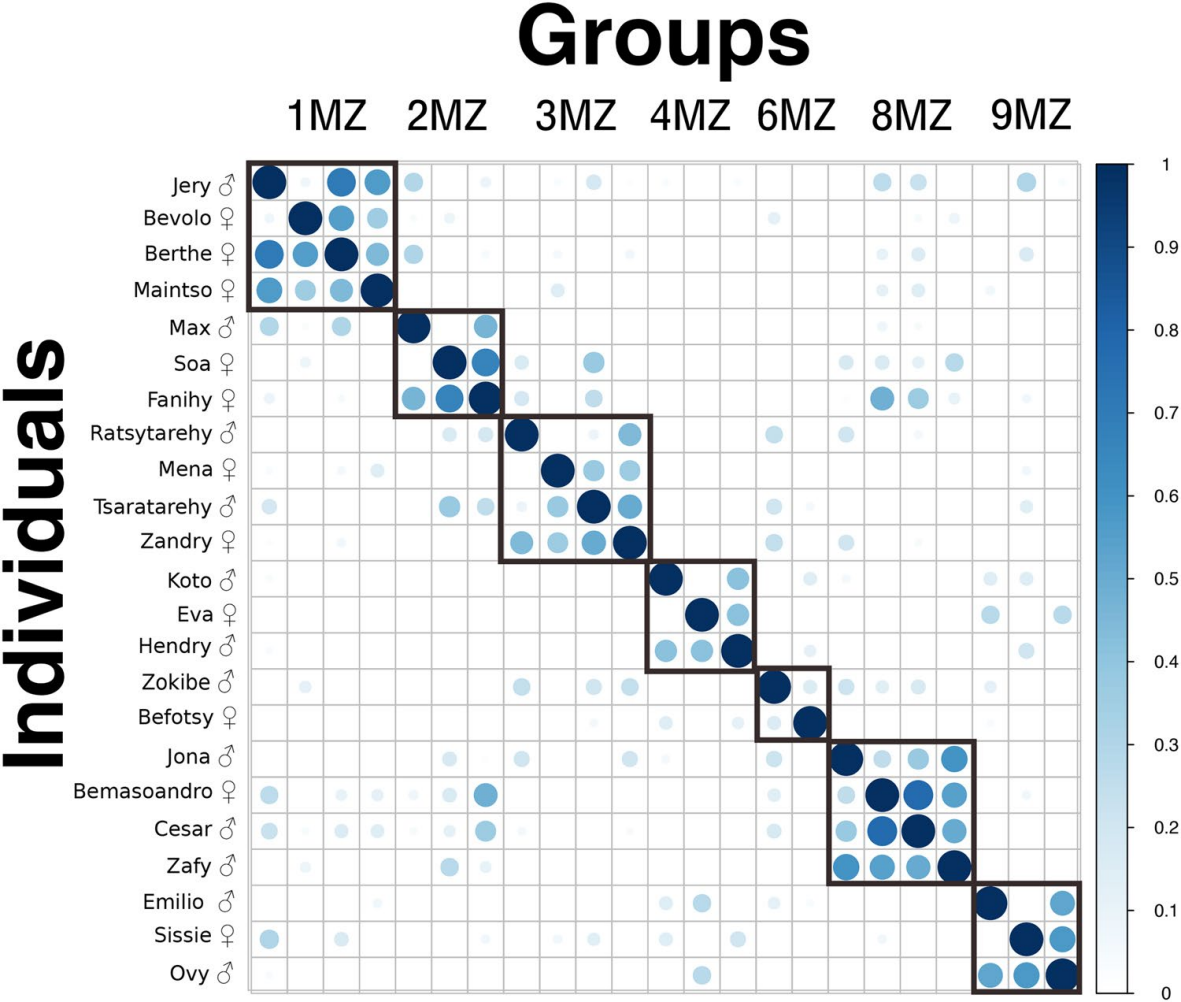
Plot of the *trioml* estimator^75^ showing the genetic relatedness among the individuals in the study groups of the indris of Maromizaha (see also Supplementary Table 3). Individual names and sexes are shown on the vertical axis; group composition is shown on the horizontal axis. Dot size and color refer to genetic relatedness: the darker and bigger the dot, the more genetically related are the individuals. The correlation plot was generated using the R package *corrplot* ^82^.

We found a variable degree of genetic variation within the groups of indris we sampled, confirming that each social group consisted of a male, an unrelated female, and their offspring (Fig. 1, Supplementary Table S5). When analyzing whether genetic distances had an effect on the temporal and frequency characteristics of DP2 at the population level, Mantel tests revealed a significant positive effect for temporal but not for frequency parameters (Table 1). For DP3, we did not find a significant effect either for temporal or frequency parameters (Table 1). Since male and female phrases are sexually dimorphic^25^, we replicated the Mantel tests separately for each sex. We found that combining the sexes together was underestimating the effect of genetic distance on male song dissimilarity, which showed a highly significant value for the temporal characteristics of DP2 (Table 1). We did not find any significant p-values for DP3 temporal and frequency features (Table 1). While there was a tendency for frequency parameters to be related to genetic relatedness in males, Mantel tests revealed a non-significant correlation between acoustics and genetics among females for DP2 and DP3 (Table 1).

**Table 1:** Results of the Mantel tests analyzing the correlation between acoustic similarity for the temporal (temp.) and frequency (freq.) parameters of descending phrases DP2 and DP3 and genetic relatedness.

		MALES			FEMALES			OVERALL		
		N	R	P-VALUE	N	R	P-VALUE	N	R	P-VALUE
DP2	Temp.	12	0.306	0.004	11	0.119	0.201	23	0.119	0.034
	Freq.		0.058	0.309		0.077	0.309		0.072	0.136
DP3	Temp.	12	0.164	0.080	10	0.006	0.473	22	0.062	0.171
	Freq.		0.172	0.055		0.015	0.234		0.094	0.074

The genetic similarity of adults ranged between 0-0.30, 0.09-0.77 for parent-offspring, and 0.11-0.50 for siblings. When we compared the pairwise acoustic indices of the categories father-daughter, father-son, mother-daughter, and mother-son to those of unrelated adults-offspring pairs, we found significant correlations of fathers and sons for the temporal and frequency parameters of DP2 and DP3 (Table 2), showing that similarity was higher within phrases emitted by related than unrelated male indris. We have also found that similarity was higher within phrases emitted by mothers and daughters than unrelated females, but only for the frequency parameters of DP2.

**Table 2:** Results of the Mantel tests analyzing the correlation between acoustic similarity for the temporal (temp.) and frequency (freq.) parameters of descending phrases DP2 and DP3 and kin information.

			SONS			DAUGHTERS		
			N	R	p	N	R	p
DP2	Temp.	FATHERS	7	0.397	0.010	7	0.033	0.410
		MOTHERS	7	0.126	0.295	7	0.227	0.114
	Freq.	FATHERS	7	0.281	0.019	7	0.026	0.533
		MOTHERS	7	0.152	0.191	7	0.311	0.038
DP3	Temp.	FATHERS	7	0.509	0.010	5	0.221	0.200
		MOTHERS	7	−0.001	0.486	5	0.115	0.200
	Freq.	FATHERS	7	0.342	0.010	5	0.059	0.500
		MOTHERS	7	0.183	0.171	5	0.001	0.500

### Individual signature and group membership

We investigated whether the variation of the DPs was consistent with the individual ID and the group membership. Despite an overall within-sex structural similarity between the DPs recorded, we found a remarkable similarity at the individual level, which reflected in the high rates of correct classification of the permuted Discriminant Function Analysis (pDFA) (Table 3). DP2s and DP3s could be assigned to the individual with accuracy greater than chance using both temporal and frequency parameters (Table 3). These results were confirmed when we repeated the analyses separating sexes (Table 3).

**Table 3:** Percentage of correctly assigned descending phrases DP2 and DP3 to the individuals, overall and by sex (N = 23 for DP2, of which N = 12 for males and N = 11 for females, N = 22 for DP3, of which N = 12 for males and N = 10 for females), and groups (group memb., N = 7) for the indris of Maromizaha.

				TRAINING	P-VALUE	TESTING	P-VALUE
DP2	INDIVIDUALITY	Temporal	Overall	44.03	0.001	24.07	0.001
			Males	51.64	0.024	29.23	0.001
			Females	50.50	0.003	37.30	0.001
		Frequency	Overall	46.62	0.001	19.59	0.001
			Males	53.56	0.046	18.15	0.002
			Females	56.39	0.001	32.13	0.001
	GROUP MEMB.	Temporal		41.47	0.475	31.09	0.258
		Frequency		45.13	0.380	29.98	0.225
DP3	INDIVIDUALITY	Temporal	Overall	81.02	0.001	28.73	0.001
			Males	91.83	0.009	35.92	0.001
			Females	71.40	0.001	45.91	0.001
		Frequency	Overall	73.50	0.001	27.79	0.001
			Males	83.00	0.009	23.04	0.001
			Females	70.47	0.001	44.60	0.001
	GROUP MEMB.	Temporal		56.04	0.443	35.56	0.125
		Frequency		56.89	0.325	33.72	0.269

In contrast, indris produced phrases that did not signal group membership accurately. In fact, pDFAs using the temporal and frequency parameters of DP2 and DP3 showed statistically significant p-values neither during the training, nor during the testing (Table 3).

## Discussion

The results presented here come from the first intra-population analysis of indris comparing the acoustic characteristics of song phrases and genetic relatedness. Using this approach, we demonstrated that the structure of males’ phrases transmits information about relatedness more consistently than females’ ones, and that song phrases possess the potential to provide conspecifics with a cue to individual identity of the emitter in both sexes. Male song-phrases DP2 transmit information about relatedness in the form of time parameters, but this information is not encoded in female calls. We found a tendency for encoding of relatedness information in the frequency structure of male song-phrases DP3. We also found that similarity of both temporal and frequency parameters in male phrases DP2 and DP3 correlate significantly correlate with genetic distance in the ‘father-son’ category. A result that for female phrases is limited only to the frequency parameters of DP2. We did not find significant correlations across sexes. Overall, we can confirm our prediction that the indris’ song phrases contain information about genetic relatedness as it has been found in other primate species (e.g.: *Mandrillus sphinx* ^14^). It is interesting to notice that the encoding of this information is strong in the temporal characteristics of the descending phrases when analyzed using genetic similarity indices, but significantly stronger in both temporal and frequency parameters of DP2 and DP3 when compared between related and unrelated males. The correlation between temporal patterns and genetic relatedness is especially interesting in the light of those studies that investigated how hybrids differed from their parental species in the acoustic characteristics of their utterances^38, 39^. In gibbons and lemurs, pulse structure and rhythmic characteristics have a particular relevance in the discrimination between hybrids and parental species^21, 38^. Our results are in agreement with analyses of intra-population variation on ground squirrels (*Urocitellus beldingi*) showing that individuals produce calls more similar to their relatives than to unrelated individuals^40^. We extended these findings showing that, in indri, relatedness is also encoded by frequency parameters of both DP2 and DP3 in males, and DP2 in females.

While the vast amount of data about primate kin recognition is devoted to the highly complex social groups of African monkeys^41, 42^, Kessler and colleagues^43^ demonstrated that the advertisement calls for the grey mouse lemur (*Microcebus murinus*) possess patrilineal signatures that mediate paternal kin recognition. Male vocal signatures have been indicated as an important mechanism for inbreeding avoidance^43^. This mechanism may have sense in the light of the long-term pair bonding of indris^44^, but may also play a role in a scenario in which extra-pair copulation can potentially contribute to increasing levels of genetic diversity within a population^45^. Moreover, even though physical fights between individuals of neighboring groups are rare in indris^46^, they involve primarily males and always include choral vocal displays^47^. Thus, song similarity between related males may mediate kin recognition and de-escalate aggressions. From a different perspective, the descending phrases are paradigmatic examples of vocal emissions with remarkable frequency modulation^23^. It is therefore possible that those phrases are acoustically more flexible and less genetically-determined. This interpretation can explain only part of our results, but it is interesting to notice that in the study by Lemasson and colleagues^48^ the genetic similarity between Campbell’s monkey females (*Cercopithecus campbelli campbelli*) did not explain the acoustic similarities of their contact calls. A higher degree of acoustic plasticity within females is supported by previous research on baboons (*Papio cynocephalus ursinus* ^49^), Campbell’s monkeys (*Cercopithecus campbelli campbelli* ^50^), and Japanese macaques (*Macaca fuscata* ^51^). Whether social factors may play a role in the acoustic plasticity of female indris need further investigation, but there is evidence that the contribution of the temporal structure to the song is less genetically determined in females than in males. Previous studies have found that females change the duration of their song to partially overlap with males’ singing^29^. Females may also adjust duration according to the number of males in their social group, while males tend to avoid overlapping each other^25, 26^.

The lack of knowledge about dispersal patterns in the indris prevents further speculation regarding the relatively higher genetic signature in male calls. In socially monogamous species there is a tendency towards female-biased dispersal^52^ that data from the field do not support for the indris. Data collected over 14 years and in different forests suggest that both male and female indris disperse (Giacoma, *unpublished data*), although dispersal frequency and distance are currently not available.

Individual variation in the vocal signals is a precondition for individual recognition, which can result in both affiliative and aggressive situations^53^. Previous research has demonstrated that sex differences may override individual differences^25^, but the results of the present study complement those findings showing that strong individuality is nevertheless encoded in the indris’ phrases. The presence of an individual signature is confirmed by both the training and testing phase of the permuted Discriminant analysis and it is valid for both temporal and frequency parameters. Our findings are in agreement with previous studies that have reported a strong individual signature in the acoustic signals of social mammals (e.g.; yellow-bellied marmots^54^; Speckled ground squirrels^32, 33^). The individuality encoding was also found in the Cao Vit gibbon male phrases^55^ and in the Bornean gibbon female great calls^56^, two species that emit songs like the indris. Our analyses suggest that individuality is encoded in both males and females, tracing an interesting path for future research in other singing primates. While most of the previous research on the indris’ song suggests that the temporal parameters play a major role in the sex-specific encoding of the vocal emissions^25, 29^, we found that also frequency parameters have potential for individual recognition. This evidence expands the recent findings of Gamba and colleagues^25^ about a sex-specific difference in pitch patterns during the song. In general, both time and frequency variables appeared to play a role in encoding individuality as they probably do for sex-specificity.

Behavioral observations support our results that singing in indris may facilitate the exchange of identity information in the context of distant communication^26, 46^. This idea is in agreement with previous studies on other primates, where long distance vocalizations were found to be useful for identifying individuals^55^ and estimating male fighting ability^57^. Although we do not have data in support of the hypothesis that song may be useful to estimate individuals’ fighting ability, our behavioral observations suggest that they indris can vocally discriminate individuals. Two lines of evidence support this idea. Torti and colleagues^26^ showed that songs elicited regrouping of particular individuals within a group. Bonadonna and colleagues^45^ observed that a female that has just been involved in extra-pair copulation did not join the song of her group mates, which were singing at a distance. We found support for the hypothesis that the use of song phrases to broadcast individuality may be essential during pair formation at distance^26^ and for the regulation of territorial spacing, where other communicative signals may be ineffective^58^.

When we analyzed the dissimilarity of phrases emitted by members of a social group, we found that pDFA could assign neither DP2 nor DP3 to the group with a classification rate higher that those predicted by chance. This result is in contrast to the findings of Knörnschild and colleagues on greater sac-winged bats (*Saccopteryx bilineata* ^59^) and Vester and colleagues on pilot whales (*Globicephala melas* ^60^), where differences of vocalizations within social groups were significantly lower than intergroup differences. The work on the greater sac-winged bats is particularly interesting because the authors found that pups modified their emissions during ontogeny and learned their songs through vocal imitation of their harem males, independently of their genetic relatedness^59^. Studies on apes and monkeys suggested a consistent degree of acoustic plasticity in nonhuman primate calls, which was observed in particular after social changes^61, 62^ or during vocal interactions^63^.

A previous investigation on the rhythmic structure of the indris’ song showed that the structure of the descending phrases did not change significantly during ontogeny^25^. Combined with our finding that group membership cues do not appear to be encoded in the phrase structure, these two elements seem to demonstrate that the indris’ song has limited flexibility when compared to other animals’ utterances and that learning may play a secondary role in song acquisition. Our results disagree with previous findings by Baker-Medard and colleagues^27^ that found significant differences in chorus songs between three groups in the Analamazaotra Reserve. There can be multiple reasons for these different findings. First, the number of songs and the number of groups considered by Baker-Medard and colleagues^27^ is smaller than those we used in the present study, possibly reducing the variation of the acoustic measurements and leading to an intergroup dissimilarity that we were not able to find. Second, it is possible that the fact that Baker-Medard and colleagues^27^ considered all the units in the songs for their analysis contributed some essential trait for group discrimination that is lacking when only the DPs are considered. However, we think that considering the most common DP types occurring in the songs should allow a proper evaluation of the acoustic variability exhibited by this species. As for the individual discrimination, we lack any evidence that the indris make similar discriminations regarding groups. However, according to our data, the potential for group recognition appears weaker than that for individual recognition. We can hypothesize that the song may play a role in the numerical assessment of group size, as McComb and colleagues^64^ have demonstrated on the African lions (*Panthera leo*). Future playbacks of the indris’ songs may improve our knowledge about the amount of information encoded by the songs.

Overall, our results confirm that vocal signals can be shaped by both genetic factors and social experience in the indris. Even in primates that emit songs with complex temporal and frequency patterns, phrases contain information about genetic relatedness and subtle variation in the acoustic structure may play a role in providing conspecifics with cues for kin identification and individual discrimination.

## Methods

### Observations and recordings

**Figure 2:**
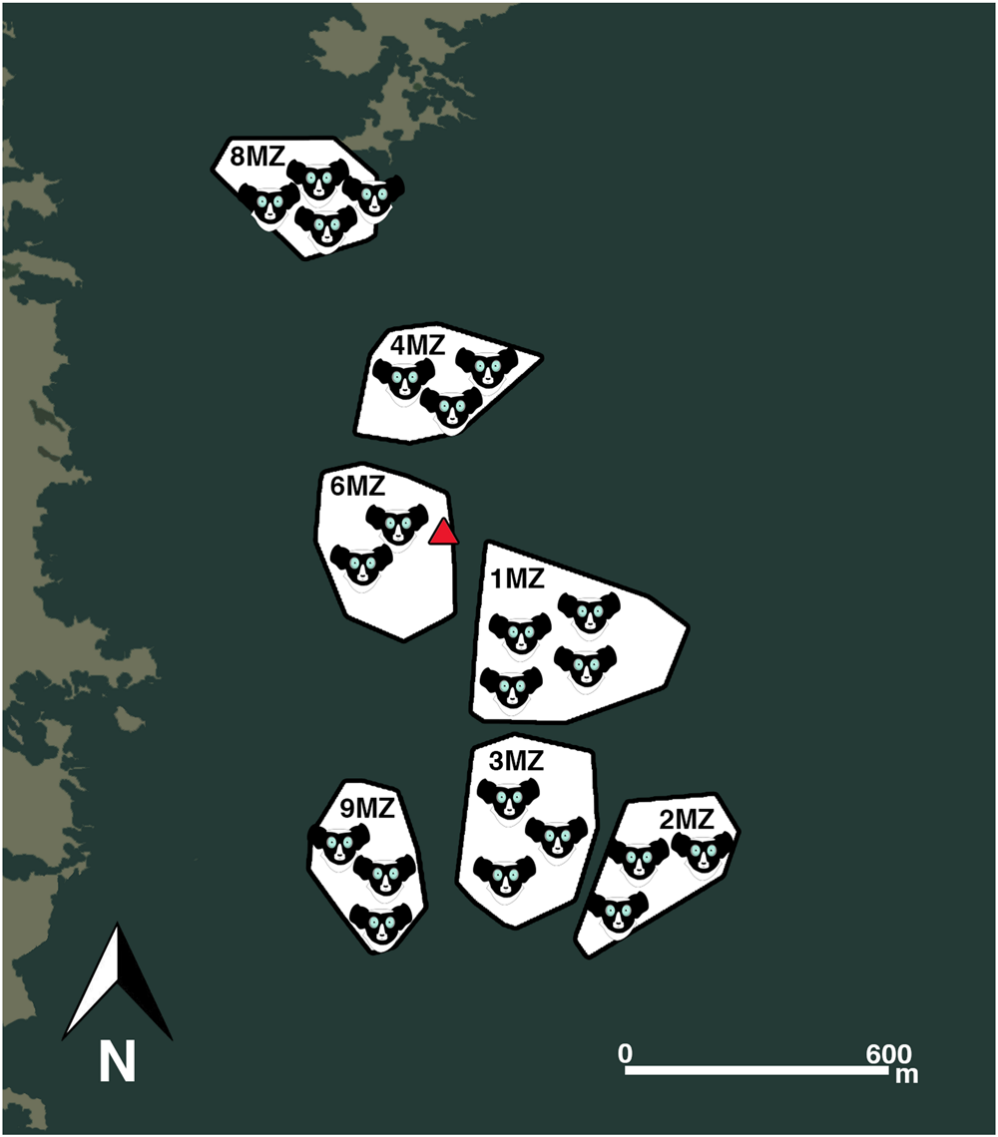
Map of the study area in the Maromizaha Forest. Minimum Convex Polygons (MCP) generated with ArcGIS 9.1 (ESRI) correspond to the 2016 home range of the study groups. Group ID is reported onto each MCP, and the indris’ face icons indicate the number of animals per group. The red shape indicates the geographical location of the Maromizaha Research Center (18°58′34.06″S 48°27′53.88″E). Drawings by Dr. Valeria Torti.

**Figure 3:**
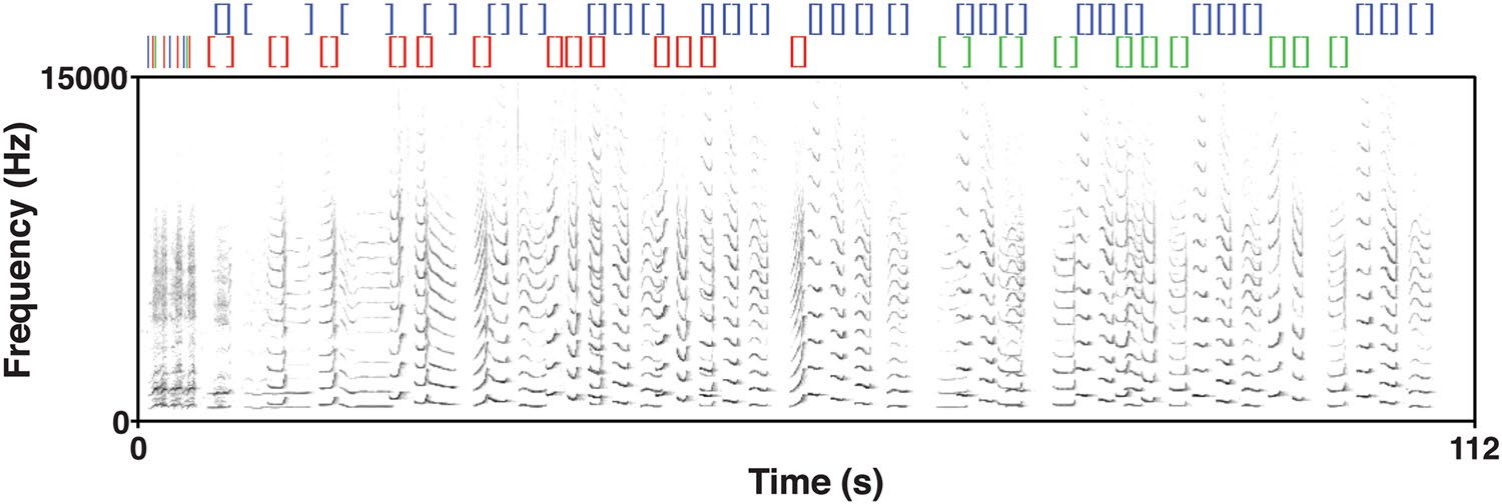
Spectrogram of the indris’ song generated using PRAAT. In this song recorded in the Maromizaha Forest, a reproductive pair is singing with a female offspring (Group 2MZ). At the top of the spectrogram, the color brackets indicate the start (“[”) and the end (“]”) of each male’s units (in blue), reproductive female’s (in red), and female offspring’s (in green).

**Figure 4:**
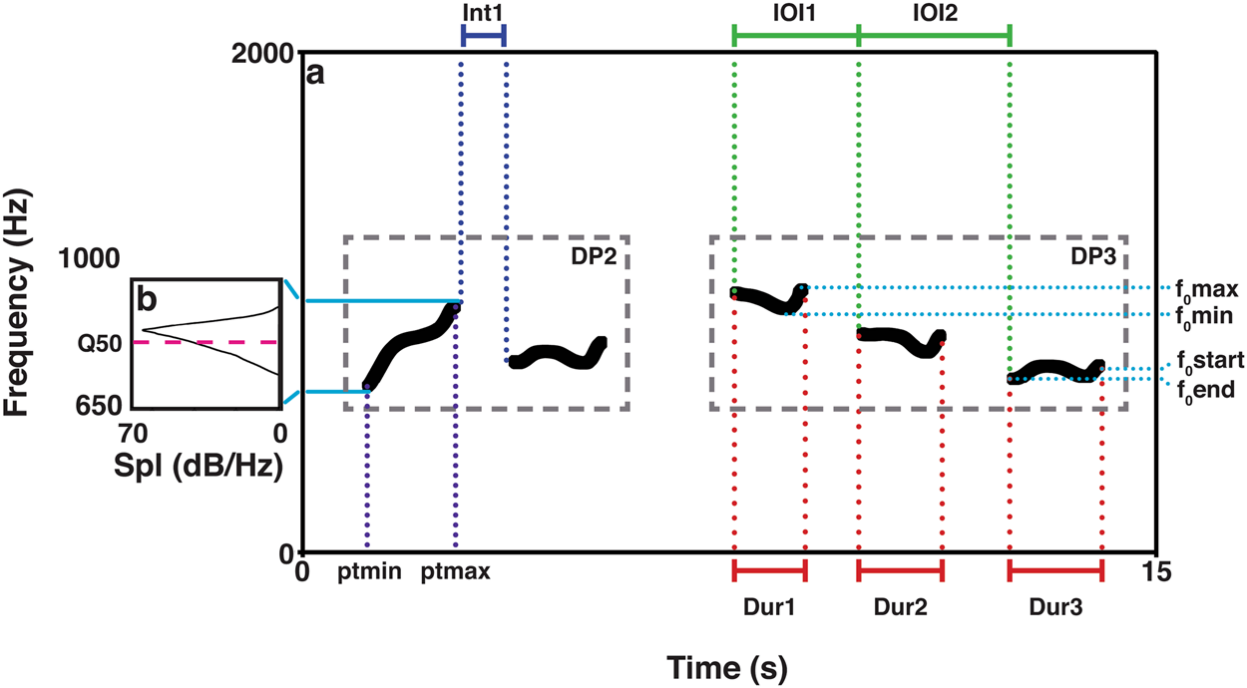
Schematic representation of the spectrogram of the isolated fundamental frequency of two descending phrases, a DP2 and a DP3 (**a**). The sound spectrogram displays time (seconds) on the x-axis, frequency (Hz) on the vertical axis. We describe acoustic parameter collection of unit duration (in red); intervals (in blue); inter-onset intervals (in green); percentage of time to the minimum (ptmin) and maximum of pitch (ptmax, in purple); maximum and minimum pitch (f_0_max and f_0_min), fundamental frequency at the beginning and at the end of a unit (f_0_start, f_0_end, in light blue). In the spectrum (**b**), the fuchsia dotted line marks the frequency corresponding to the upper limit of the second quartile of energy in the spectrum (Q50). The sound spectrum displays sound pressure level (Spl/dB) on the x-axis, frequency on the vertical axis.

We studied seven groups living in the Maromizaha Forest (18°56′49′′S, 48°27′53′′E). We collected data in the field from 2011 to 2016, for a total of 24 months. We observed a social group per day, approximately from 6 AM to 1 PM. We identified the indris individually thanks to natural marks. Group composition ranged from 2 to 4 indris (Fig. 2; details are provided in Table 4). Recordings were made using solid-state recorders (SoundDevices 702, Olympus S100 and LS05, and Tascam DR-100, DR-40, and DR-05) equipped with Sennheiser (ME 66 and ME 67) or AKG (CK 98) shotgun microphones. The microphone signal was recorded at a sampling rate of 44.1 kHz, 16 bit. We recorded all the songs at a distance comprised between 2 and 20 m, keeping visual contact with the vocalizing animals (Fig. 3). We made all efforts to orientate the microphone toward the focal uttering individuals. All recordings were carried out without the use of playback stimuli, and nothing was done to modify the behavior of the indris. When in the field, we had one observer per individual in a group. Using the focal animal sampling technique^65^, we were able to attribute each vocalization to a signaler. From the individual song contributions, we extracted 1066 descending phrases consisting of two units (hereafter, DP2), and 1259 descending phrases consisting of three units (DP3; Fig. 4). We focused on DP2s and DP3s because they are the most common phrase types in the indris’ song^25^. The sampling included phrases emitted by seven males (of which six sired at least one offspring), seven siring females, 9 offspring (five males and four females, Table 4). We included in our analyses only those individuals contributing at least four DPs. Our final sample included 23 individuals for the DP2s and 22 individuals for the DP3s.

**Table 4:** Summary of group, ID, sex and year of birth of offspring.

Group	ID	Sex/YOB
1MZ	Jery	♂
	Bevolo	♀
	Maintso	♀ 2010
	Berthe	♀ 2012
2MZ	Max	♂
	Soa	♀
	Fanihy	♀ 2012
3MZ	Ratsytarehy	♂
	Mena	♀
	Tsaratarehy	♂ before 2009
	Zandry	♀ 2010
4MZ	Koto	♂
	Eva	♀
	Hendry	♂ before 2009
6MZ	Zokibe	♂
	Befotsy	♀
8MZ	Jona	♂
	Bemasoandro	♀
	Cesar	♂ before 2009
	Zafy	♂ 2012
9MZ	Emilio	♂
	Sissie	♀
	Ovy	♂ 2013

For each group, the reproductive pair is listed first. The year of birth is not reported for the reproductive pair.

### Genetic analyses

The genetic analysis confirmed the identification of the reproductive pairs we independently derived from our behavioral observations^25, 26^. We collected fecal samples from 23 individuals and we stored all the samples in RNAlater® Ambion^66^ at room temperature in the field and at 4 °C in the laboratory.

### DNA extraction

We extracted genomic DNA from feces using the QIAamp DNA® Stool Mini Kit (Qiagen®, Hilden, Germany) with the following changes from the manufacturer’s protocol (QIAamp DNA Stoll Handbook 04/2010). We used 300 mg stool; we added 35 μl of proteinase K and incubated at 70° Celsius for 30 minutes during the purification phase. We applied 75 μl Buffer AE on the QIAamp membrane for the first DNA elution and incubated the spin column with Buffer AE at room temperature for 15 minutes.

For the samples collected in 2014, we used the automated robotic workstation QIAcube HT supported by the software QIAxtractor 4.17.1 (Qiagen®) to conduct DNA purification. We set the protocol for QXT Liquid DNA V1. First, we bathed the 2.0 mL tubes containing 300 mg of smashed feces and 1.6 mL of Buffer ASL at 70 °C for at least 5 minutes. After that, tubes were centrifuged at 13000 RPM for 10 minutes. We transferred 200 μl of supernatant to separate wells of the QIAextractor lysis plate and we started the run. At the end of the process, we obtained 70 µl of DNA elution for each sample. We stored the extracted DNA at 4 °C for immediate use.

### DNA genotyping

We selected a set of 6 microsatellite marker loci identified as potentially variable in indri that provided good quality amplification products for multiplex PCRs^67^ (Table 5). A fluorescent dye (FAM, HEX) labeled the 5′ end forward primer of each locus to analyze simultaneously loci of similar allelic size. PCR amplification was carried out in 10 µL reaction volume containing: 5 µL Multiplex PCR Master Mix (Qiagen®), 0.1 µm of each primer, 2 µL DNA template, 2 µL RNase-free water. We set the cycle conditions as follows: a pre-incubation step at 95 °C for 15 min; 50 cycles with denaturation at 94 °C for 30 s, annealing at 54 °C or 60 °C (depending on the locus, see Table 5) for 90 s. The first extension phase was at 72 °C for 60 s; the final extension phase at 60 °C for 30 min.

**Table 5:** Microsatellite loci used in this study, with respective primers, and annealing temperatures. The number of PCR cycles is 50.

Locus	Forward primer	Reverse Primer	Repeat motif	Annealing temp. (°C)	Size range (bp)
67HDZ25	GGACCCTAATTCAAATATCACCTC	GGCATTTCTACTCCAGGTTGG	(CA)_16_	54	218-253
67HDZ62	AGCCCTTTCTCTCAATGCC	CCTTCTTTGTTATCTTTCTGCATC	(GT)_21_	54	203-217
67HDZ18	GGACTGGTAGATTTCTGGGTTTAG	CAGCCACTCCAATGCAAAG	(CA)_7_C(CA)_15_	60	164-190
67HDZ55	TCAGGAGTTGGGACCAGGG	ATGAAGGGATGGAGGTGGG	(GT)_18_	60	312-334
67HDZ180	TCCCCTCCTCAGTCATTTCTC	CGTGAAGCTCGTGTGTATGG	(CA)_17_	60	113-136
67HDZ39	CAGAGCCAGGGTTCAAATTC	TTGTCTTTTCTGCCACTGTAGG	(CA)_11_	60	148-162

We estimated the allele size by electrophoresis using a 48-capillary ABI 3730 DNA Analyzer (Applied Biosystems). We mixed 1 µL of PCR product with 6.85 µL HiDi formamide (Applied Biosystems) and 0.15 µL Genescan 500-ROX size standard (Applied Biosystems). We conducted automated allele calling using the software GENEMAPPER 3.7 (Applied Biosystems). We then confirmed by eye all the allele sizes and checked for consistency across replicate PCRs of the same sample or from the same individual for a certain locus (minimum three replicates for heterozygotes and five replicates for homozygotes).

### Relatedness analysis

We estimated relatedness among individuals using the R package *related* ^68^. First, we compared seven different relatedness estimators commonly used in the literature, five moment estimators^69–73^ and two likelihood-based estimators, the dyadic likelihood estimator – *dyadml* ^74^ and the triadic likelihood estimator – *trioml* ^75^. Using the allele frequencies observed in our dataset, we simulated datasets of 100 pairs for four known relatedness categories (parent–offspring, full-sibling, half-sibling, and unrelated). We chose the *trioml* ^75^ estimator to calculate relatedness for all possible dyadic combinations because it showed the highest consistency and obtained a matrix in which the more positive the index, the more two individuals are genetically related. Since indri lives in family group^28, 47^, the historical record of group composition since 2009 allowed us to infer parental relationships among individuals based on behavioral observations, especially between mother and offspring. We were able to assign a social father to each of the offspring included in the study. To define parental information for the comparison of acoustic distances, we run paternity analyses including as potential fathers all the adult males sampled (Bonadonna, unpublished data) and could assign paternity for nine offspring (out of 10).

### Acoustic and statistical analyses

Because the singers’ phrases could overlap each in the temporal and frequency domain, we first extracted the fundamental frequency using a manual procedure and then obtained the pitch contour using a semi-automatic process in Praat^76^. We then added 0.5 s of silence at the beginning and the end of each phrase. Because each unit within a phrase went through the same set of measurements, we collected a minimum of 10 measurements in the temporal domain and a minimum of 20 measurements of pitch variability for each DP. The complete list of variables we measured is in Supplementary Table S6, while some parameters are presented in Fig. 4. Further details about the methodology used can be found in Gamba and colleagues^25^.

We used principal components analysis (Factor analysis in IBM SPSS 24.0.0.1) to reduce the data to uncorrelated principal components (PCs) using separately temporal and frequency measurements of DP2s and DP3s. We obtained four PCs exceeding eigenvalue 1 for the temporal measurements and five for frequency variables of DP2s. We obtained six PCs exceeding eigenvalue 1 for each of the two sets of variables of DP3s. To understand whether genetic relatedness could explain acoustic similarities, we transformed the PCs obtained for each of these sets in a Euclidean distance matrix (function *dist* in R 3.2.3) and then calculated the average individual means. We then run the Mantel tests (9999 randomizations^77, 78^) on the average individual means against the matrix of genetic indices (package *vegan* in R^79^). All matrix indices were normalized to have a value between 0 and 1 before entering the analyses.

We used the Mantel test to evaluate whether the acoustic distance differed among individuals paired by categories (‘father-daughter’; ‘mother-daughter’, ‘father-son’, ‘mother-son’). We assessed the correlation between the acoustic similarity matrix and a binary matrix indicating the category (e.g.; ‘father-daughter’; refs 77 and 80) for each pair of song phrases. A significant correlation would indicate a difference in the similarity of phrases given by one of the pairs mentioned above (e.g.; father-daughter) when compared with phrases emitted by unrelated reproductive adults (e.g.; other ‘fathers’ in the sample) and offspring (e.g.; ‘daughters’ of other pairs).

To understand whether we could identify a potential for individual recognition or group membership we submitted the component scores to permutated linear discriminant function analysis^81^ in R (using a custom script by Roger Mundry). When testing for individual differences, we used the individual as test factor and the song from which the DPs were extracted as a control factor. We also ran the analyses split by sex. When testing for group membership, we used the group as test factor and the individual identity as control factor. We split all the analyses into two phases, a training phase and a testing phase (R. Mundry, personal communication) for which we collected the correct classification rate and the p-value.

### Data availability

Data and programs used for the analyses presented in the paper are available to the Editorial board members and the referees upon request or already included in the Supporting Information.

## Electronic supplementary material

Supplementary Information
