# Supplementary material for: An intra-population analysis of the indris’ song dissimilarity in the light of genetic distance
Source: Sci Rep. 2017 Aug 31;7:10140. doi: 10.1038/s41598-017-10656-9 (PMC5579264; doi:10.1038/s41598-017-10656-9)
Supplement: Supplementary file 1 — Supplementary Information [file 41598_2017_10656_MOESM1_ESM.pdf]

An intra-population analysis of the indris' song dissimilarity in the light of genetic distance.

Valeria Torti[1‡], Giovanna Bonadonna[1‡], Chiara De Gregorio[1], Daria Valente[1], Rose Marie Randrianarison[2,3], Olivier Friard[1], Luca Pozzi[4], Marco Gamba[1†*], Cristina Giacoma[1†]

[1] *Department of Life Sciences and Systems Biology (DBIOS), University of Torino, Italy*

[2] *Département de Paléontologie et d'Anthropologie Biologique, Faculté des Sciences, Université d'Antananarivo, Antananarivo, Madagascar.*

[3] *Groupe d'étude et de recherche sur les primates de Madagascar (GERP), Antananarivo, Madagascar*

[4] *Department of Anthropology, The University of Texas at San Antonio, One UTSA Circle, San Antonio, TX 78249, United States of America.*

[‡] *joint primary author*

[†] *joint senior author*

[*] *to whom correspondence should be addressed, postal address:*

Department of Life Sciences and Systems Biology,

University of Torino,

10134 Torino

Italy

Supplementary Table S1 Factor loadings for temporal parameters of DP2 on the first four principal components.

	PARAMETER	*COMPONENT 1*	*COMPONENT 2*	*COMPONENT 3*	*COMPONENT 4*
**DP2 TEMPORAL PARAMETERS**	IOI1	0.957	-0.132	0.088	0.107
	Dur_Unit1	0.833	0.288	0.435	0.146
	Dur_Int1	0.757	-0.498	-0.281	0.03
	Dur_Unit2	0.688	0.547	0.151	-0.392
	PtMin_Unit1	0.478	0.454	-0.447	0.217
	PtMax_Unit2	0.337	-0.674	0.179	-0.185
	PercDur_Unit1	0.021	0.352	0.731	0.505
	PercDur_Unit2	-0.179	0.786	0.109	-0.55
	PtMin_Unit2	-0.334	0.395	-0.246	0.501
	PtMax_Unit1	-0.407	-0.4	0.646	-0.093

Supplementary Table S2 Factor loadings for frequency parameters of DP2 on the first five principal components.

	PARAMETER	*COMPONENT 1*	*COMPONENT 2*	*COMPONENT 3*	*COMPONENT 4*	*COMPONENT 5*
**DP2 FREQUENCY PARAMETERS**	$f_0$mean_Unit2	0.889	0.276	-0.339	0.007	0.041
	$f_0$mean_Unit1	0.874	-0.395	0.19	-0.154	0.031
	Q75_Unit1	0.864	-0.362	0.107	-0.259	0.086
	Q50_Unit1	0.857	-0.386	0.102	-0.249	0.083
	Q25_Unit1	0.849	-0.409	0.098	-0.328	0.08
	Q50_Unit2	0.849	0.211	-0.436	-0.1	0.07
	Q75_Unit2	0.849	0.192	-0.436	-0.103	0.078
	Q25_Unit2	0.848	0.228	-0.434	-0.096	0.06
	$f_0$min_Unit2	0.813	0.273	-0.413	0.005	0.008
	$f_0$max_Unit1	0.812	0.011	0.478	-0.043	-0.058
	$f_0$max_Unit2	0.76	0.389	0.041	0.355	0.122
	$f_0$start_Unit1	0.664	0.573	0.066	0.338	-0.236
	$f_0$start_Unit2	0.664	0.573	0.066	0.338	-0.236
	$f_0$min_Unit1	0.656	-0.683	0.084	0.189	-0.087
	$f_0$end_Unit1	0.51	-0.735	0.145	0.286	-0.07
	$f_0$end_Unit2	0.51	-0.735	0.145	0.286	-0.07
	$f_0$abs_slope_Unit1	0.444	0.518	0.673	-0.1	-0.094
	$f_0$abs_slope_Unit2	0.444	0.518	0.673	-0.1	-0.094
	$f_0$rangeratio_Unit2	0.021	0.058	0.215	0.446	0.845
	$f_0$rangeratio_Unit1	0.009	0.464	0.296	-0.401	0.345

Supplementary Table S3 Factor loadings for temporal parameters of DP3 on the first six principal components.

	PARAMETER	*COMPONENT 1*	*COMPONENT 2*	*COMPONENT 3*	*COMPONENT 4*	*COMPONENT 5*	*COMPONENT 6*
**DP3 TEMPORAL PARAMETERS**	IOI2	0.896	0.257	-0.232	-0.118	-0.178	-0.015
	Dur_Int2	0.849	-0.186	-0.331	-0.138	-0.256	-0.176
	IOI1	0.835	0.24	0.35	0.218	0.127	-0.144
	Dur_Int1	0.801	-0.149	0.399	-0.105	0.322	0.028
	Dur_Unit2	0.58	0.753	0.001	-0.041	0.003	0.212
	PercDur_Int2	0.557	-0.591	-0.41	-0.126	-0.351	-0.128
	Dur_Unit1	0.556	0.566	0.166	0.482	-0.13	-0.273
	PtMax_Unit3	0.512	-0.141	-0.341	0.133	0.101	0.404
	PtMax_Unit2	0.36	-0.275	0.225	0.466	0.429	0.025
	PercDur_Int1	0.297	-0.626	0.533	-0.088	0.375	0.178
	PtMin_Unit1	0.207	0.115	0.608	-0.338	-0.312	-0.075
	Dur_Unit3	0.204	0.808	-0.115	-0.288	0.299	-0.231
	PercDur_Unit2	-0.098	0.724	0.048	0.027	-0.072	0.537
	PtMin_Unit2	-0.138	0.073	0.413	0.066	-0.453	0.403
	PtMax_Unit1	-0.139	-0.067	-0.555	0.566	0.217	0.069
	PercDur_Unit1	-0.315	0.202	0.249	0.789	-0.288	-0.209
	PtMin_Unit3	-0.391	-0.168	0.429	-0.07	-0.077	-0.235
	PercDur_Unit3	-0.593	0.568	-0.13	-0.305	0.329	-0.196

Supplementary Table S4 Factor loadings for frequency parameters of DP3 on the first six principal components.

	PARAMETER	*COMPONENT 1*	*COMPONENT 2*	*COMPONENT 3*	*COMPONENT 4*	*COMPONENT 5*	*COMPONENT 6*
**DP3 FREQUENCY PARAMETERS**	$f_0$min_Unit2	0.897	-0.029	-0.11	-0.355	-0.172	0.068
	Q50_Unit2	0.878	-0.062	-0.22	-0.358	-0.099	0.03
	Q75_Unit2	0.878	-0.078	-0.224	-0.35	-0.088	0.025
	Q25_Unit2	0.876	-0.047	-0.213	-0.365	-0.11	0.036
	$f_0$mean_Unit2	0.818	-0.463	0.239	-0.036	0.074	-0.077
	Q75_Unit1	0.812	-0.455	0.172	-0.164	0.049	-0.092
	Q50_Unit1	0.81	-0.461	0.166	-0.154	0.043	-0.097
	Q25_Unit1	0.808	-0.466	0.163	-0.142	0.039	-0.099
	$f_0$min_Unit2	0.796	-0.046	-0.23	-0.314	-0.288	0.051
	$f_0$max_Unit1	0.784	-0.174	0.426	-0.025	0.195	-0-064
	$f_0$mean_Unit3	0.768	0.449	-0.371	0.194	0.116	-0.001
	$f_0$max_Unit2	0.739	0.045	0.144	-0.316	-0.312	0.241
	$f_0$min_Unit3	0.721	0.377	-0.426	0.099	0.064	0.04
	Q25_Unit3	0.716	0.348	-0.476	0.179	0.262	-0.035
	Q50_Unit3	0.713	0.348	-0.476	0.184	0.272	-0.042
	Q75_Unit3	0.709	0.333	-0.475	0.188	0.279	-0.048
	$f_0$min_Unit1	0.597	-0.635	0.162	0.397	-0.059	0.039
	$f_0$max_Unit3	0.533	0.604	0.131	0.276	-0.33	-0.14
	$f_0$end_Unit1	0.513	-0.613	0.159	0.541	0.04	0.023
	$f_0$end_Unit2	0.513	-0.613	0.159	0.541	0.04	0.023
	$f_0$end_Unit3	0.513	-0.613	0.159	0.541	0.04	0.023
	$f_0$start_Unit1	0.475	0.708	0.178	0.354	-0.116	0.068
	$f_0$start_Unit2	0.475	0.708	0.178	0.354	-0.116	0.068
	$f_0$start_Unit3	0.475	0.708	0.178	0.354	-0.116	0.068
	$f_0$abs_slope_Unit1	0.343	0.462	0.741	-0.182	0.188	0.014
	$f_0$abs_slope_Unit2	0.343	0.462	0.741	-0.182	0.188	0.014
	$f_0$abs_slope_Unit3	0.343	0.462	0.741	-0.182	0.188	0.014
	$f_0$rangeratio_Unit3	0.043	0.243	0.234	0.191	-0.511	-0.473
	$f_0$rangeratio_Unit1	-0.031	0.246	0.039	-0.348	0.381	-0.4
	$f_0$rangeratio_Unit2	-0.056	0.068	0.157	-0.011	0.072	0.73

Supplementary Table S5 Matrix of genetic relatedness (*trioml* estimator) among the individuals of the study groups of the indris of Maromizaha.

	befotsy	bemasoandro	berthe	bevolo	cesare	emilio	eva	fanihy	hendry	jery	jonah	koto	maintso	max	mena	ovy	ratsytarehy	sissie	soa	tsaratarehy	zafy	zandry	zokybe
**befotsy**	1	0	0	0	0	0.038	0	0	0.114	0	0	0.142	0	0	0	0	0	0	0	0.051	0	0	0.155
**bemasoandro**	0	1	0.101	0	0.77	0	0	0.483	0	0.268	0.252	0	0.111	0.076	0	0	0	0.06	0.169	0	0.54	0	0.139
**berthe**	0	0.101	1	0.55	0.153	0	0	0.043	0	0.709	0	0	0.449	0.302	0.053	0	0	0.167	0	0	0	0.063	0
**bevolo**	0	0	0.55	1	0.04	0	0	0	0	0.07	0	0	0.359	0.032	0	0	0	0.007	0.088	0	0.089	0	0.118
**cesare**	0	0.77	0.153	0.04	1	0	0	0.361	0	0.222	0.375	0	0.147	0.04	0	0	0.053	0	0.111	0	0.5	0.04	0.173
**emilio**	0.038	0	0	0	0	1	0.274	0	0.003	0	0	0.148	0.068	0	0	0.525	0	0	0	0	0	0	0.118
**eva**	0	0	0	0	0	0.274	1	0	0.417	0	0	0	0	0	0	0.277	0	0	0	0	0	0	0
**fanihy**	0	0.483	0.043	0	0.361	0	0	1	0	0.088	0.027	0	0	0.465	0	0	0.18	0.061	0.665	0.25	0.105	0	0
**hendry**	0.114	0	0	0	0	0.003	0.417	0	1	0.037	0	0.417	0	0	0.005	0	0	0.203	0	0	0	0	0
**jery**	0	0.268	0.709	0.07	0.222	0	0	0.088	0.037	1	0	0.037	0.566	0.293	0.037	0.037	0	0.304	0	0.188	0	0.026	0
**jonah**	0	0.252	0	0	0.375	0	0	0.027	0	0	1	0.057	0	0	0	0	0.204	0	0.172	0	0.595	0.204	0.212
**koto**	0.142	0	0	0	0	0.148	0	0	0.417	0.037	0.057	1	0	0	0	0	0	0.149	0	0	0	0	0
**maintso**	0	0.111	0.449	0.359	0.147	0.068	0	0	0	0.566	0	0	1	0	0.148	0	0	0	0	0	0	0	0
**max**	0	0.076	0.302	0.032	0.04	0	0	0.465	0	0.293	0	0	0	1	0	0	0	0	0	0	0	0	0
**mena**	0	0	0.053	0	0	0	0	0	0.005	0.037	0	0	0.148	0	1	0	0	0.063	0	0.375	0	0.36	0
**ovy**	0	0	0	0	0	0.525	0.277	0	0	0.037	0	0	0	0	0	1	0	0.578	0	0	0	0	0
**ratsytarehy**	0	0	0	0	0.053	0	0	0.18	0	0	0.204	0	0	0	0	0	1	0	0.164	0.08	0	0.445	0.248
**sissie**	0	0.06	0.167	0.007	0	0	0	0.061	0.203	0.304	0	0.149	0	0	0.063	0.578	0	1	0	0.134	0	0	0
**soa**	0	0.169	0	0.088	0.111	0	0	0.665	0	0	0.172	0	0	0	0	0	0.164	0	1	0.375	0.276	0	0
**tsaratarehy**	0.051	0	0	0	0	0	0	0.25	0	0.188	0	0	0	0	0.375	0	0.08	0.134	0.375	1	0	0.5	0.206
**zafy**	0	0.54	0	0.089	0.5	0	0	0.105	0	0	0.595	0	0	0	0	0	0	0	0.276	0	1	0	0
**zandry**	0	0	0.063	0	0.04	0	0	0	0	0.026	0.204	0	0	0	0.36	0	0.445	0	0	0.5	0	1	0.248
**zokybe**	0.155	0.139	0	0.118	0.173	0.118	0	0	0	0	0.212	0	0	0	0	0	0.248	0	0	0.206	0	0.248	1

Supplementary Table S6 List and abbreviations of the acoustic parameters.

PARAMETER	*DESCRIPTION*
Dur_Unit(x)	Total duration of the unit, measured in seconds
PercDur_Unit(x)	Unit's duration percentage related to the total phrase length
$f_0$mean_Unit(x)	Mean fundamental frequency value across the unit (Hz)
$f_0$max_Unit(x)	Maximum fundamental frequency value across the unit (Hz)
$f_0$min_Unit(x)	Minimum fundamental frequency value across the unit (Hz)
$f_0$start_Unit(x)	Frequency value of f_0 and the start of the unit
$f_0$end_Unit(x)	Frequency value of f_0 and the end of the unit
ptmax_Unit(x)	Time % occurring from the unit beginning up to the maximum f_0, assuming that the unit duration is 100
ptmin_Unit(x)	Time % occurring from the unit beginning up to the minimum f_0, assuming that the unit duration is 100
$f_0$abs_slope_Unit(x)	Decline of the unit f_0 over time (Hz/s)
FM_rate	Number of complete cycles of f_0 modulation per second (s^{-1})
$f_0$rangeratio_Unit(x)	Range between maximum and minimum f_0
Q25_Unit(x)	Frequency values at the upper limit of the first quartiles (Q25%) of energy
Q50_Unit(x)	Frequency values at the upper limit of the second quartiles (Q50%) of energy
Q75_Unit (x)	Frequency values at the upper limit of the third quartiles (Q75%) of energy
IOI1	Inter-onset interval for the first and the second unit
IOI2	Inter-onset interval for the second and the third unit
Dur_int(x)	Duration of the interval between units
PercDur_Int(x)	Interval's percentage duration related to the phrase total length
